# High seroprevalence of *Toxoplasma gondii *infection in a subset of Mexican patients with work accidents and low socioeconomic status

**DOI:** 10.1186/1756-3305-5-13

**Published:** 2012-01-11

**Authors:** Cosme Alvarado-Esquivel, Alejandro Torres-Castorena, Oliver Liesenfeld, Sergio Estrada-Martínez, Jesús D Urbina-Álvarez

**Affiliations:** 1 Faculty of Medicine and Nutrition, Juárez University of Durango State. Avenida Universidad S/N. 34000 Durango, Dgo, Mexico; 2 Mexican Social Security Institute, Avenida Normal # 200, 34000, Durango City, Durango, Mexico; 3 Institute for Microbiology and Hygiene, Campus Benjamin Franklin, Charité Medical School, Hindenburgdamm 27. D-12203 Berlin, Germany. Present address: Roche Molecular Diagnostics, Pleasanton, CA. USA; 4 Institute for Scientific Research, Juárez University of Durango State. Avenida Universidad S/N. 34000 Durango, Dgo. Mexico

**Keywords:** *Toxoplasma gondii*, seroprevalence, work accidents, case-control study, epidemiology

## Abstract

**Background:**

*Toxoplasma gondii *has been associated with reflex impairment and traffic accidents. It is unknown whether *Toxoplasma *infection might be associated with work accidents. Therefore, using a case-control seroprevalence study design, 133 patients with a recent work accident and 266 control subjects of the general population from the same region were examined with enzyme-linked immunoassays for the presence and levels of anti-*Toxoplasma *IgG antibodies and anti-*Toxoplasma *IgM antibodies. Socio-demographic, work, clinical and behavioral characteristics from each worker were obtained.

**Results:**

Eleven (8.3%) of 133 patients, and 14 (5.3%) of 266 controls had anti-*T. gondii *IgG antibodies. Anti-*T. gondii *IgG levels were higher than 150 IU/ml in 8 (6%) patients and 10 (3.8%) controls. Anti-*T. gondii *IgM antibodies were found in one (0.8%) of the workers, and in 6 (2.3%) of the controls. No statistically significant differences in the IgG seroprevalences, frequencies of high IgG levels, and IgM seroprevalences among patients and controls were found. In contrast, a low socio-economic level in patients with work accidents was associated with *Toxoplasma *seropositivity (*P *= 0.01). Patients with work accidents and low socioeconomic status showed a significantly (OR = 3.38; 95% CI: 0.84-16.06; *P *= 0.04) higher seroprevalence of *T. gondii *infection than controls of the same socioeconomic status (15.1% vs. 5%, respectively). Multivariate analysis showed a positive association of *T. gondii *infection with boar meat consumption (OR = 3.04; 95% CI: 1.03-8.94; *P *= 0.04). In contrast, a negative association between *T. gondii *infection and national trips (OR = 0.40; 95% CI: 0.17-0.96; *P *= 0.04), sausage consumption (OR = 0.20; 95% CI: 0.05-0.68; *P *= 0.01), and ham consumption (OR = 0.16; 95% CI: 0.05-0.51; *P *= 0.002) was found.

**Conclusions:**

In the study described here seropositivity to *T. gondii *was associated to work accidents in a subset of patients with low socioeconomic status. This is the first report of an association of *T. gondii *infection and work accidents. Further studies to confirm our results are needed. Results may help in designing optimal prevention strategies to avoid *T. gondii *infection.

## Background

The parasite *Toxoplasma gondii (T. gondii) *infects humans and animals worldwide [1]. Major routes of infection with *T. gondii *include eating undercooked or raw meat containing tissue cysts, and ingesting food or water contaminated with oocysts [1,2]. The clinical spectrum of infection with *T. gondii *in humans varies from asymptomatic latent infection to severe disease affecting eyes, lymph nodes and central nervous system [1,2]. Infection with *T. gondii *has been linked to traffic accidents. Fukunaga et al. [3] reported a rare case of brain calcification supposedly caused by infection with *T. gondii *in a man that had a fatal traffic accident. Gyori et al. [4] reported an unusual case of cerebral toxoplasmosis leading to a fatal vehicular crash. In retrospective case-control studies, Flegr et al. [5], Yereli et al. [6], and Kocazeybek et al. [7] found significantly higher seroprevalences of *T. gondii *infection in subjects with traffic accidents than those in control groups. The increased risk of traffic accidents in *Toxoplasma*-infected subjects was confirmed in a prospective cohort study [8]. These results and the fact that *T. gondii *may cause visual [1,2] and reflex impairments [9,10] in humans, and changes in behavior in rodents [11] and humans [12] lead us to raise the question whether *T. gondii *infection is linked to work accidents too. Since there is no information about the association of *T. gondii *infection with work accidents we used a matched seroprevalence case control study to determine the association of *T. gondii *seropositivity and anti-*T. gondii *IgG levels with work accidents. In addition, we investigated socio-demographic, work, clinical, and behavioral characteristics associated with *T. gondii *seropositivity.

## Methods

### Study design and study populations

Through a case-control seroprevalence study, 133 patients with a recent work accident and 266 control subjects of the general population from the same region were examined with enzyme-linked immunoassays for the presence and levels of anti-*Toxoplasma *IgG antibodies and for the presence of anti-*Toxoplasma *IgM antibodies. This study was performed from January 2009 to December 2010 in workers attending the Department of Occupational Medicine in a public hospital (Mexican Social Security Institute) in Durango City, Mexico. Inclusion criteria for cases were: 1) to have had a recent work accident regardless its severity; 2) aged 17 years and older; 3) any gender; and 4) who accepted to participate in the study. As a strategy for recruiting workers we invited them when seeking medical consultation and incapacity in the Department of Occupational Medicine. Of 160 patients invited, 133 (83.1%) agreed to participate in the study. All patients resided in Durango. Eighty one patients were male and 52 were female. The mean age of the patients was 33.75 ± 11.04 years (range: 17-60 years). The control group consisted of 266 subjects drawn from the general population of Durango City and matched with cases by age, gender, and residence. The mean age in controls was 33.36 ± 11.03 (range: 17-60) and did not differ significantly from that of patients with a recent work accident (*P *= 0.99).

### Ethical aspects

This study was approved by the Institutional Ethical Committee of the Mexican Social Security Institute. The purpose and procedures of the study were explained to all participants, and a written informed consent was obtained from all of them.

### Socio-demographic, clinical, work, and behavioral data

We explored the characteristics of the participants with the aid of a standardized questionnaire. Socio-demographic data including age, gender, occupation, birth place, residence, educational level and socioeconomic status were included in the analysis. Work data included seniority (number of years) in the work place, type of accident (falls, slipping, trips), cause of accident, type of injury, work shift, time of day the accident happened, history of alcohol or drug consumption just before the accident, and number of previous work accidents in life. Contributing and confounding risk factors of behavioral data from all participants were also obtained. These factors included animal contacts, presence of cats at home or in the neighborhood, cleaning cat feces, travelling in Mexico and abroad, meat consumption (pork, beef, goat, sheep, boar, chicken, turkey, rabbit, venison, squirrel, horse, or other), degree of meat cooking, consumption of unpasteurised milk, dried or cured meat (ham, sausages, salami, chorizo, or dried beef), unwashed raw vegetables, fruits, or untreated water, frequency of eating out of home (in restaurants or fast food outlets), and contact with soil (gardening or agriculture). Questions regarding contributing and confounding risk factors of behavioral data from all participants refer to ever during life. Clinical data included presence of underlying disease, history of blood transfusion or transplant, and memory, reflex, hearing, and visual impairments.

### Laboratory tests

Serum samples were obtained from all participants and kept frozen at -20°C until analyzed. Serum samples were assayed by qualitative and quantitative methods for anti-*T. gondii *IgG antibodies with a commercially available enzyme immunoassay: "*Toxoplasma *IgG" kit (International Immuno-Diagnostics, Foster City, California). Anti-*T. gondii *IgG antibody levels were expressed as International Units (IU) per ml, and a result equal to or greater than 8 IU/ml was considered positive. In addition, sera positive for *T. gondii *IgG were further tested for anti-*T. gondii *IgM antibodies by a commercially available enzyme immunoassay: "*Toxoplasma *IgM" kit (International Immuno-Diagnostics, Foster City, California). All tests were performed following the instructions of the manufacturer.

### Statistical Analysis

Results were analyzed with the aid of the software Epi Info version 3.5.1 and SPSS 15.0 (SPSS Inc. Chicago, Illinois). Age among the groups was compared by the student's *t *test. For comparison of the frequencies among the groups, the Yates corrected test and when indicated the Fisher exact test with 1-tailed *P *value and exact confidence limits were used. Bivariate and multivariate analyses were used to evaluate the association between the characteristics of the subjects and *T. gondii *infection. The association of *T. gondii *infection with work accidents was assessed by comparing the seroprevalences of anti-*T. gondii *IgG antibodies in patients with work accidents and controls. In addition, the association of *T. gondii *infection with specific accidents (falls, slipping, trips) was assessed by comparing the seroprevalences of anti-*T. gondii *IgG antibodies in patients with and patients without a specific accident. Seropositivity to IgG or IgM was analyzed with dichotomous data. Variables were included in the multivariate analysis if they had a *P *value equal to or less than 0.20 in the bivariate analysis. Age-adjusted odd ratio (OR) and 95% confidence interval (CI) were calculated by multivariate analysis using multiple, unconditional, logistic regression. A *P *value less than 0.05 was considered statistically significant.

## Results

**Figure 1:**
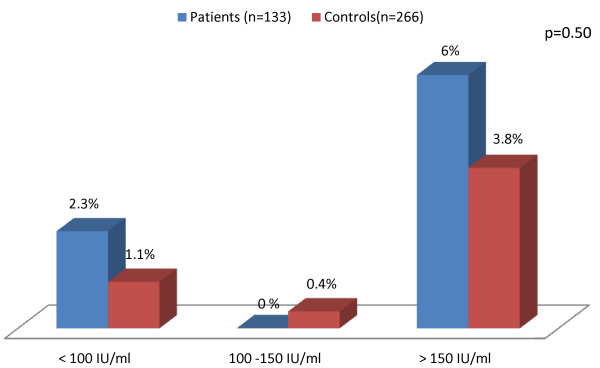
**Frequency of anti-*T. gondii *IgG antibody levels in patients and controls**. Anti-*T. gondii *IgG antibody levels were similar in patients and controls.

Eleven (8.3%) of the 133 patients, and 14 (5.3%) of the 266 controls were positive for anti-*T. gondii *IgG antibodies (OR = 1.62; 95% CI: 0.71-3.68; *P *= 0.17). Anti-*T. gondii *IgG levels were higher than 150 IU/ml in 8 (6%) patients and 10 (3.8%) controls (OR = 1.63; 95% CI: 0.63-4.25; *P *= 0.21) (Figure 1). Anti-*T. gondii *IgM antibodies were found in one (0.8%) of the patients, and in 6 (2.3%) of the controls (OR = 0.45; 95% CI: 0.07-2.71; *P *= 0.26).

General socio-demographic characteristics of subjects with work accidents are shown in Table 1. Of the socio-demographic characteristics, only a low socio-economic level was associated with *Toxoplasma *seropositivity (OR = 5.71; 95% CI: 1.33-24.54; *P *= 0.01). Patients with work accidents and low socioeconomic level showed a significantly (OR = 3.38; 95% CI: 0.84-16.06; *P *= 0.04) higher seroprevalence (8/53: 15.1%) of *T. gondii *infection than control subjects (4/80: 5.0%) of the same socioeconomic level.

**Table 1:** Socio-demographic characteristics of patients with work accidents and their association with *Toxoplasma *infection.

			Prevalence of		
			*T. gondii *infection		
				
Characteristic	**No**.	*%*	**No**.	*%*	*P *value
Age groups (years)					
30 or less	60	45.1	3	5	0.37
31-50	60	45.1	6	10	
51-70	13	9.8	2	15.4	
Gender					
Male	81	60.9	9	11.1	0.2
Female	52	39.1	2	3.8	
Occupation					
Construction	7	5.3	2	28.6	0.48
Carpenter	3	2.3	0	0	
Driver	9	6.8	0	0	
Electrician	1	0.8	0	0	
Employee	62	46.6	3	4.8	
Cattle raising	1	0.8	0	0	
Smith	1	0.8	0	0	
Cleaning	5	3.8	0	0	
Machinist	5	3.8	1	20	
Factory worker	7	5.3	2	28.6	
Machine operator	1	0.8	0	0	
Professional	1	0.8	0	0	
Other	30	22.6	3	10	
Residence area					
Urban	124	93.2	9	7.3	0.2
Suburban	3	2.3	1	33.3	
Rural	6	4.5	1	16.7	
Educational level					
No education	6	4.5	2	33.3	0.07
1-12 years	127	95.5	9	7.1	
Socio-economic level					
Low	53	39.8	8	15.1	0.01
Medium	78	58.6	2	2.6	

Bivariate analysis of *T. gondii *infection and work characteristics including seniority in the work, type of accident, cause of accident, type of injury, work shift, time of day the accident happened, history of alcohol or drug consumption just before the accident, and number of previous work accidents in life did not show any statistically significant association.

Clinical characteristics as evidence for the presence of underlying disease, history of blood transfusion or transplant, memory, reflex, hearing, and visual impairments did not show any association with *Toxoplasma *seropositivity.

Behavioral characteristics with a *P *value equal to or less than 0.20 in the bivariate analysis included the presence of cats at home, raising animals, travelling in Mexico and abroad, consumption of goat, boar, and turkey meats, consumption of raw meat, unpasteurized goat milk, unwashed raw vegetables, sausages, ham, salami and untreated water; eating out of home, and soil floors at home. Multivariate analysis of these characteristics showed a positive association of *T. gondii *infection with boar meat consumption (OR = 3.04; 95% CI: 1.03-8.94; *P *= 0.04), and a negative association of *T. gondii *infection with national trips (OR = 0.40; 95% CI: 0.17-0.96; *P *= 0.04), sausage consumption (OR = 0.20; 95% CI: 0.05-0.68; *P *= 0.01), and ham consumption (OR = 0.16; 95% CI: 0.05-0.51; *P *= 0.002) (Table 2).

**Table 2:** Multivariate analysis of selected characteristics of participants and their association with *Toxoplasma gondii *infection.

	Age-adjusted	95% confidence	*P*
Characteristic	Odds ratio	interval	value
Cats at home	1.46	0.64-3.33	0.35
Raising animals	1.55	0.56-4.28	0.39
Travel abroad	0.47	0.17-1.32	0.15
National trips	0.40	0.17-0.96	0.04
Goat meat consumption	1.96	0.85-4.52	0.11
Boar meat consumption	3.04	1.03-8.94	0.04
Turkey meat consumption	0.67	0.29-1.56	0.36
Raw meat consumption	5.00	0.45-55.4	0.19
Raw goat milk consumption	2.77	0.72-10.57	0.13
Sausage consumption	0.20	0.05-0.68	0.01
Ham consumption	0.16	0.05-0.51	0.002
Salami consumption	0.40	0.15-1.05	0.06
Unwashed raw vegetables	0.45	0.13-1.56	0.20
Untreated water consumption	2.35	0.91-6.08	0.07
Eating out of home	0.59	0.18-1.87	0.37
Soil floors at home	2.11	0.66-6.7	0.20

## Discussion

We found comparable seroprevalences of both anti-*T. gondii *IgG and IgM antibodies, and comparable frequencies of anti-*T. gondii *IgG levels higher than 150 IU/ml in patients suffering from a recent work accident and controls. However, a subset of workers with work accidents and low socioeconomic level showed a significantly higher seroprevalence of *T. gondii *infection than control subjects of the same socioeconomic level. These results suggest that in general, *T. gondii *infection seems not be associated with work accidents in the population of Mexican workers from Durango; but in particular, suggest that *T. gondii *infection might be a contributing factor for work accidents in workers with low socioeconomic status. In principle, *T. gondii *may affect senses important to avoid any kind of accidents namely vision and hearing. It is well known that infections with *T. gondii *may lead to chorioretinitis [1,2] thus affecting vision. Furthermore, infection with *T. gondii *has been linked to deafness [13] or hearing impairment [14]. Seroprevalence of *T. gondii *has been found higher in individuals with reflex [9,10,15] and memory [16] impairments than those without these impairments. In the present study, we did not find an association of *T. gondii *infection with a specific impairment in patients with work accidents. This fact might explain the lack of association between *T. gondii *infection and work accidents in general.

Of the socio-demographic characteristics, a low socio-economic status was associated with *T. gondii *infection in our patients. This result confirms that of a previous report in our region [17]. A low socioeconomic level may be linked to malnutrition, and this factor might impair the host defense against *T. gondii *infection. Therefore, it is likely that health could be more easily impacted by *T. gondii *in workers with low socioeconomic status than in workers with higher socioeconomic status. This is supported by the fact that health in Tepehuanos, one of the poorest populations in Durango, has been impacted by *T. gondii *infection. In a recent study in Tepehuanos, seropositivity to *T. gondii *was associated with frequent headaches and hearing impairment [14]. In addition, other factors might contribute to an increase in the likelihood of an accident in *T. gondii *seropositive subjects. Several reports indicate that *T. gondii *infection may affect the reaction time in infected individuals. Havlícek et al. [18] in a double blind study showed significantly longer reaction times of subjects with latent toxoplasmosis in comparison with those of controls. Moreover, those researchers found a positive correlation between length of infection and mean reaction time suggesting that slow and cumulative effects of latent toxoplasmosis rather than a one-step (and possible transient) effect of acute toxoplasmosis disease are responsible for the decrease of psychomotor performance of infected subjects. Novotná et al. [19] showed that heterozygous men with both the RhD plus and the RhD minus alleles were protected against prolongation of reaction times caused by infection with *T. gondii*. In a further study in men and women, Flegr et al. [20] confirmed that RhD-positive subjects were less sensitive to the influence of latent toxoplasmosis on reaction times than RhD-negative subjects. In the present study, Rh blood typing in patients and controls was not determined.

Behavioral/mental disorders in humans including schizophrenia, mood disorders, personality changes, and cognitive impairments may be related to infection with *T. gondii *[12,21,22]. In addition, experimental infection with *T. gondii *in rodents has caused changes in behavior including a loss in aversion to cat odor [11]. Behavioral/mental changes induced by *T. gondii *might impair the work performance leading to work accidents.

With respect to behavioral characteristics, multivariate analysis showed a positive association of *T. gondii *infection with consumption of boar meat. Remarkably, in a previous independent study, we also found and association of boar meat consumption with *T. gondii *infection [23].

Our study has limitations. First, the sample size and power may not have been large enough to determine all risk factors and associations. Second, the type of accidents could be not severe enough to find further associations. The general population in Durango has shown low seroprevalence of *T. gondii *infection [23,24] as compared with those in other Mexican states [24]. A low seroprevalence in Durango has been found even when using various kits detecting anti-*T. gondii *IgG antibodies. For instance, Velasco-Castrejón et al. [24] reported a 9.6% seroprevalence in population of Durango by an indirect immunofluorescence antibody test. Similarly, a 6.1% seroprevalence was found in pregnant women who attended a public hospital of Durango City by a microparticle enzyme immunoassay [25]. Therefore, the seroprevalences found in the present study by enzyme-linked immunosorbent assay seem to be reliable. It is likely that the dry climate and high altitude in Durango may partially explain the low prevalence in Durango. Infection with *T. gondii *is more prevalent in low-lying areas than in mountainous areas and in humid areas than in dry areas [1].

## Conclusions

In the study described here seropositivity to *T. gondii *was associated to work accidents in a subset of patients with low socioeconomic status. This is the first report of an association of *T. gondii *infection and work accidents. Further studies with a larger sample size will have to confirm the findings presented here. The identification of a behavioral characteristic associated with *T. gondii *seropositivity will help to design optimal preventive measures against *T. gondii *infection.

## Competing interests

The authors declare that they have no competing interests.

## Authors' contributions

CAE conceived and designed the study protocol, participated in the coordination and management of the study, applied the questionnaires, performed the laboratory tests and data analysis, and wrote the manuscript. ATC and JDUA obtained clinical data, applied the questionnaires and performed the data analysis. SEM performed the statistical analysis. OL performed the data analysis, and wrote the manuscript. All authors read and approved the final version of the manuscript.
